# An Uncommon Case of Lactational Hypercalcemia With Diagnostic and Therapeutic Challenges

**DOI:** 10.7759/cureus.98247

**Published:** 2025-12-01

**Authors:** Yaline Sathiyaseelan, Ananth Srinivashan, Guganesan Vitush

**Affiliations:** 1 Department of Internal Medicine, St. Mary's Hospital, Isle of Wight National Health Service (NHS) Trust, Newport, GBR; 2 Department of General Surgery, St. Mary's Hospital, Isle of Wight National Health Service (NHS) Trust, Newport, GBR

**Keywords:** case report, ectopic secretion, lactational hypercalcemia, parathyroid hormone, postpartum hypercalcemia

## Abstract

A 35-year-old female patient was referred to General Medicine by Obstetrics for high calcium levels identified in routine postpartum lab tests. She had persistent vomiting throughout pregnancy and had been managed with antiemetics without lasting relief. On initial work-up, her parathyroid hormone (PTH) was found to be suppressed, suggesting a non-PTH-related etiology. Comprehensive investigations, including bone profile, erythrocyte sedimentation rate (ESR), myeloma screening, imaging (CT, PET), and respiratory evaluation, excluded malignancy, sarcoidosis, and other common causes. Despite a trial of intravenous (IV) fluids, low-dose steroids, and calcium management, the patient's condition persisted, necessitating referral to specialized centers. After exhaustive consultations, ectopic PTH secretion was identified as the likely cause of the elevated calcium levels. Given the challenges related to lactation and breastfeeding, the management required coordination between obstetrics, endocrinology, and maternity services to ultimately manage hypercalcemia. This case highlights the diagnostic complexity and the importance of an interdisciplinary approach in managing rare conditions like lactational hypercalcemia and ectopic PTH secretion in the postpartum period.

## Introduction

Postpartum hypercalcemia is an uncommon but clinically important condition that can present significant diagnostic and management challenges. In most adults, hypercalcemia arises from primary hyperparathyroidism or malignancy, but in the postpartum and lactation period, alternative etiologies such as parathyroid hormone-related peptide (PTHrP) excess, vitamin D metabolism disorders, and genetic defects must also be considered [1,2].

During pregnancy and lactation, calcium homeostasis undergoes complex adaptations to meet the increased calcium demands of the developing fetus and milk production. These physiologic changes involve elevated circulating levels of 1,25-dihydroxyvitamin D, increased intestinal calcium absorption, and secretion of PTHrP from the mammary glands, which enhances bone resorption to mobilize calcium for breast milk synthesis [1,3]. While these mechanisms are typically well regulated, excessive or dysregulated PTHrP production can lead to pathologic hypercalcemia in the absence of malignancy or parathyroid disease [1,3].

In addition, recent studies have identified CYP24A1 gene mutations as an emerging cause of pregnancy- and lactation-associated hypercalcemia. These mutations impair the degradation of active vitamin D metabolites, resulting in increased calcium absorption and impaired calcium clearance [2,4]. Recognition of this mechanism has expanded the differential diagnosis of postpartum hypercalcemia beyond traditional endocrine and malignant causes.

We present a rare case of postpartum hypercalcemia in a lactating woman with suppressed endogenous parathyroid hormone (PTH), in whom ectopic PTH or PTHrP secretion was suspected after exclusion of common causes. This report highlights the diagnostic complexity of postpartum hypercalcemia and emphasizes the need for a multidisciplinary approach involving obstetrics, endocrinology, and general medicine teams.

## Case presentation

**Figure 1:**
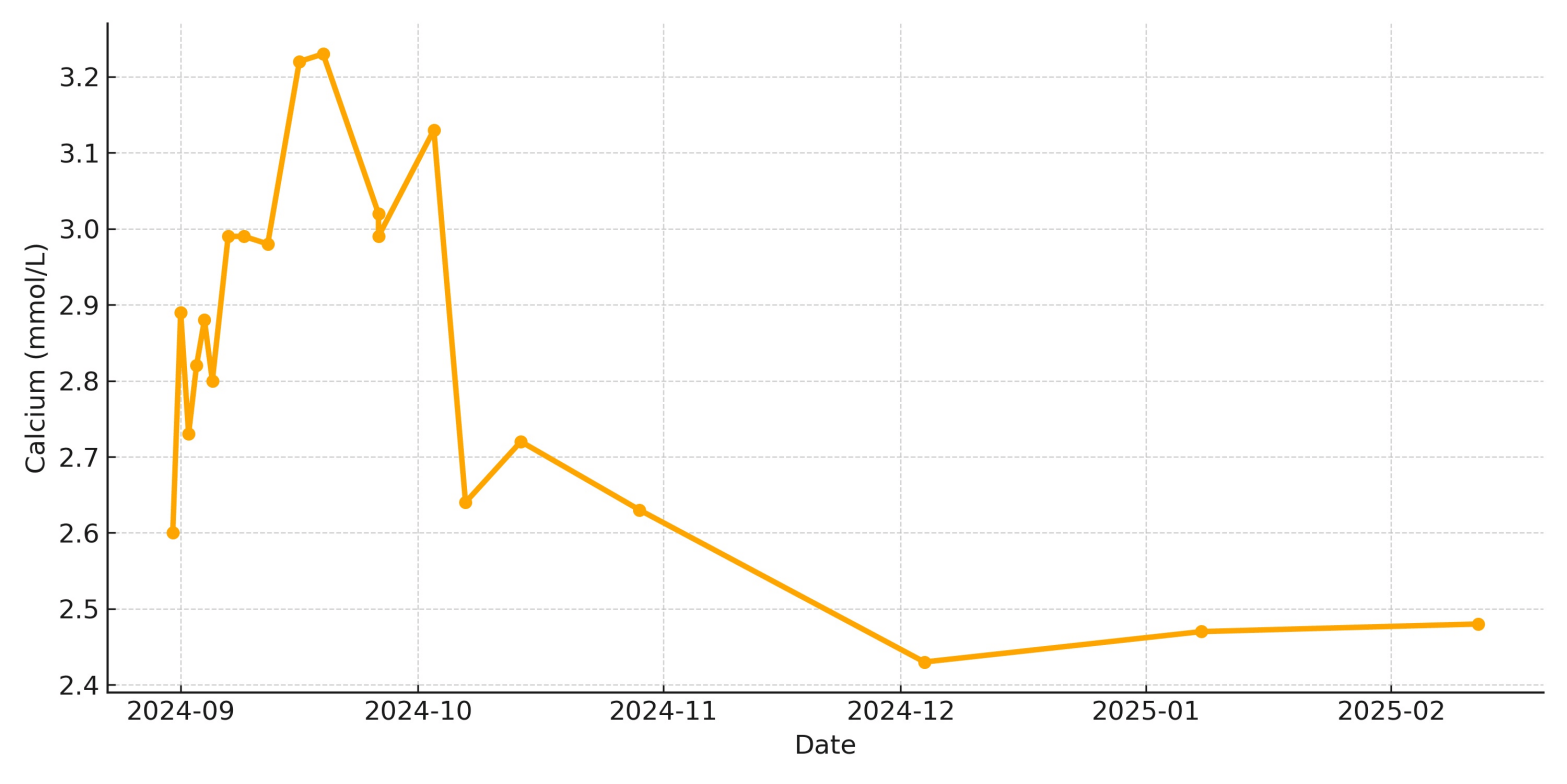
Trend of the patient's serum calcium over time

A 35-year-old woman was referred to the General Medicine service by Obstetrics after routine post-delivery laboratory investigations revealed elevated serum calcium levels (Figure 1). She had delivered normally two months ago but reported persistent vomiting throughout pregnancy, which had been managed with antiemetics with limited effect. She didn't have any typical features as "stones, bones, abdominal moans, and psychiatric groans" of hypercalcemia.

On admission, physical examination demonstrated signs of dehydration, dry mucosa, dry skin but no fever, weight loss, or other features suggestive of systemic illness or malignancy.

Investigations

Initial laboratory evaluation (Table 1) revealed hypercalcemia (corrected calcium: 2.91-3.23 mmol/L (reference range 2.2-2.6 mmol/L) with suppressed PTH at 0.7 pmol/L (reference range 1.5-6.9 pmol/L).

**Table 1:** Laboratory results MCV: Mean cell volume; ESR: erythrocyte sedimentation rate; CRP: C-reactive protein; ALT: alanine aminotransferase; ALP: alkaline phosphatase; PTH: parathyroid hormone.

Parameter	Normal Range (Units)	04-Sep-2024	05-Sep-2024	07-Sep-2024	09-Sep-2024	12-Sep-2024	16-Sep-2024	19-Sep-2024	26-Sep-2024	03-Oct-2024	07-Oct-2024	14-Oct-2024	29-Oct-2024	04-Dec-2024	08-Jan-2025	12-Feb-2025
Haemoglobin	Female: 115-150 g/L	105	110	117	121	128	129	–	114	–	–	135	125	–	–	131
MCV	80-100 fL	94.7	94.5	94.3	95.4	94.2	94.1	94.1	93.3	–	–	93.3	93.1	–	–	95.2
Platelets	150-400×10⁹/L	386	349	356	402	395	407	410	369	–	–	369	346	–	–	379
ESR	Female: <20 mm/h	–	–	–	–	–	–	–	–	–	–	–	–	–	–	5
CRP	<5 mg/L	0.3	0.6	0.7	0.5	1.0	1.3	–	0.9	–	–	0.9	–	–	–	–
Creatinine	Female: 45-90 µmol/L	91	85	80	92	87	85	75	88	87	85	85	81	–	–	79
ALT	Female: <33 U/L	13	10	9	14	15	17	–	–	11	10	–	–	–	–	8
ALP	30-130 U/L	59	58	61	70	66	75	97	81	91	105	98	107	93	80	62
Total Bilirubin	3-21 µmol/L	4	5	4	6	4	6	5	5	–	–	–	–	–	–	6
Calcium (Adjusted)	2.20-2.60 mmol/L	2.82	2.88	2.80	2.99	2.98	3.22	3.23	3.02	3.13	2.64	2.72	2.63	2.43	2.47	2.48
Phosphate	0.8-1.5 mmol/L	1.20	1.28	1.24	1.35	1.16	1.16	1.10	–	–	1.38	1.42	1.55	1.40	1.46	1.47
Vitamin D (25-OH)	>50 nmol/L (sufficient); 25–50 (insufficient); <25 (deficient)	–	–	–	–	–	–	–	–	–	–	–	–	40.8	–	25.9
Intact PTH	1.6-6.9 pmol/L	–	–	–	–	–	–	–	–	–	–	1.1	–	–	–	3.2

Imaging, including CT head and PET-CT, showed no evidence of malignancy or mass lesions, and multiple myeloma was excluded with normal immunology such as normal immunoglobulin (IgA) ,IgG, Ig M and normal CT, PET scan without any abnormal findings. Angiotensin-converting enzyme (ACE) levels were normal, making sarcoidosis less likely. Respiratory review was unremarkable. Vitamin D (25-OH) level was mild low 40.8 nmol/l and was not significant.

Management

The patient received intravenous hydration from the detection of increased serum calcium, which produced minimal improvement in calcium levels. Bisphosphonate therapy was initially withheld due to diagnostic uncertainty. Vitamin D deficiency was identified, but supplementation resulted in worsening hypercalcemia and was discontinued.

Low-dose prednisone (20 mg daily) was commenced but produced only limited biochemical response. Given the possible lactational contribution to hypercalcemia, cessation of breastfeeding was advised; however, the patient declined. Maternity services facilitated discussions regarding donor milk provision, though this was not ultimately accepted.

Endocrinology consultation recommended continuing glucocorticoid therapy for four to eight weeks with cautious tapering, aggressive fluid management, and close biochemical monitoring. Genetic testing for CYP24A1 mutation, which can impair vitamin D metabolism and contribute to postpartum hypercalcemia, was initiated; however, the results were negative.

Outcome

Calcium levels gradually trended downward with supportive measures and corticosteroid therapy, though they remained mildly elevated at the time of discharge. The patient was discharged in stable condition with instructions for adequate oral hydration, continuation of prednisone, and close outpatient follow-up with endocrinology.

## Discussion

Hypercalcemia in the postpartum period is an uncommon clinical finding and poses significant diagnostic and therapeutic challenges. If left untreated, it could cause severe complications such as arrythmias, kidney stones, kidney failure, pancreatitis, peptic ulcer diseases, bone destruction, even confusion and potential coma. 

The working differential included sarcoidosis, malignancy, vitamin D-related disorders, and drug-induced hypercalcemia. The family history of sarcoidosis (mother) raised suspicion in our case, but imaging and angiotensin-converting enzyme (ACE) levels did not support this diagnosis. The suppressed endogenous PTH in the setting of persistent hypercalcemia suggested a non-parathyroid cause, with ectopic PTH or PTHrP secretion considered. PTHrP measurement was not available locally due to resource limitations.

Our patient presented with persistent hypercalcemia in the setting of suppressed PTH, which effectively excluded primary hyperparathyroidism [1]. Initial workup excluded malignancy and sarcoidosis. The persistence of suppressed endogenous PTH raised the suspicion of ectopic PTH or PTHrP secretion. While PTHrP levels could not be measured due to resource limitations, this remains a strong possibility given the biochemical profile and clinical context [1,3].

Lactation itself is associated with increased calcium demand, driven by parathyroid hormone-related peptide secreted by mammary tissue and upregulation of bone resorption to support milk production [3]. In rare circumstances, this physiologic mechanism may become exaggerated, resulting in clinically significant hypercalcemia [1,3]. Furthermore, emerging evidence suggests that CYP24A1 mutations, which impair the degradation of active vitamin D metabolites, can predispose individuals to hypercalcemia during pregnancy and lactation [2,4]. Genetic testing was performed to explore this possibility, which yielded negative results with no evidence of a vitamin D gene defect [4].

Similar cases have been described in the literature. Kovacs et al. reported severe postpartum hypercalcemia due to excessive PTHrP secretion without evidence of malignancy, which mirrors the biochemical profile observed in our patient [5]. Likewise, studies on calcium-regulating hormones during pregnancy and lactation demonstrate that PTHrP secretion increases substantially during breastfeeding and can lead to transient hypercalcemia [3]. More recently, Schlingmann et al. highlighted the role of CYP24A1 mutations in predisposing to pregnancy- and lactation-associated hypercalcemia, underscoring the importance of genetic evaluation in such patients [6]. Our case aligns with these observations, reinforcing the need to consider both hormonal dysregulation and genetic predisposition in postpartum hypercalcemia [1-4].

Management of postpartum hypercalcemia must be individualized. Hydration, corticosteroids, and cessation of vitamin D supplementation are often first-line approaches [1,4]. Glucocorticoids decrease intestinal calcium absorption, increase urinary excretion of calcium, and interfere with Vitamin D metabolism. Glucocorticoids are used for PTHrP postpartum hypercalcemia because they suppress 1,25(OH)₂D production, increase vitamin D breakdown, reduce calcium absorption from the gut, decrease bone resorption, act quickly, and are suitable for short-term management postpartum.

Bisphosphonates, while effective, are used cautiously in lactating women due to safety concerns [1]. In this case, corticosteroid therapy and fluid management achieved gradual biochemical improvement. Breastfeeding cessation is frequently recommended, but patient preferences and psychosocial factors must be balanced against clinical risks [1,3]. Our patient declined cessation, highlighting the complexity of management in such scenarios.

This case underscores three important clinical lessons. First, suppressed PTH in the presence of hypercalcemia warrants consideration of ectopic PTH/PTHrP secretion, even in the postpartum setting [1,3]. Second, lactation-associated changes in calcium metabolism can mimic more sinister pathologies and must be carefully distinguished [3]. Finally, a multidisciplinary approach involving obstetrics, endocrinology, general medicine, and maternity services is critical for optimizing outcomes in rare and complex presentations [1-4].

## Conclusions

Postpartum hypercalcemia is a rare but potentially serious condition that requires careful evaluation and a multidisciplinary approach. This case highlights the diagnostic challenges of differentiating lactation-associated hypercalcemia from other causes, including ectopic PTH or PTHrP secretion. Suppressed endogenous PTH in the setting of elevated calcium should prompt consideration of ectopic sources, even in the postpartum period. Postpartum hypercalcemia is managed first by removing contributing factors - stopping calcium and vitamin D supplements - and considering partial weaning if lactation-related PTHrP is involved. Aggressive IV hydration is used early to increase renal calcium excretion, while glucocorticoids are often preferred because they quickly lower calcium by suppressing vitamin D activation, enhancing its breakdown, reducing intestinal absorption, and modestly decreasing bone resorption. Bisphosphonates are generally reserved for severe or refractory cases because they persist in bone for years, may interfere with normal lactation-related bone turnover, carry theoretical risks to breastfed infants and future pregnancies, and cannot be reversed once given. Corticosteroids are favored postpartum because they effectively treat vitamin D-driven hypercalcemia, do not accumulate in bone, have minimal transfer into breast milk, and are safer for short-term use, including when CYP24A1 abnormalities are present. CYP24A1 encodes the enzyme responsible for breaking down both 25-hydroxyvitamin D and its active form, 1,25-dihydroxyvitamin D, through 24-hydroxylation. When a woman has a loss-of-function CYP24A1 mutation or reduced CYP24A1 activity, this degradation pathway fails, causing excessive accumulation of active vitamin D. The elevated 1,25-dihydroxyvitamin D markedly increases intestinal calcium absorption and enhances bone resorption, while simultaneously suppressing PTH. The combined effect is persistent hypercalcemia driven by unregulated vitamin D activity. Early recognition, comprehensive investigation, and coordinated care among obstetrics, endocrinology, and general medicine teams are essential to optimize outcomes in these complex cases. Awareness of rare etiologies, such as lactational hypercalcemia and CYP24A1 mutations, can prevent prolonged diagnostic delays and guide appropriate therapeutic strategies.
